# Nitroaromatic explosives detection using electrochemically exfoliated graphene

**DOI:** 10.1038/srep33276

**Published:** 2016-09-16

**Authors:** Ying Teng Yew, Adriano Ambrosi, Martin Pumera

**Affiliations:** 1 Division of Chemistry and Biological Chemistry, School of Physical and Mathematical Sciences, Nanyang Technological University, 21 Nanyang Link, Singapore 637371, Singapore

## Abstract

Detection of nitroaromatic explosives is of paramount importance from security point of view. Graphene sheets obtained from the electrochemical anodic exfoliation of graphite foil in different electrolytes (LiClO_4_ and Na_2_SO_4_) were compared and tested as electrode material for the electrochemical detection of 2,4-dinitrotoluene (DNT) and 2,4,6-trinitrotoluene (TNT) in seawater. Voltammetry analysis demonstrated the superior electrochemical performance of graphene produced in LiClO_4_, resulting in higher sensitivity and linearity for the explosives detection and lower limit of detection (LOD) compared to the graphene obtained in Na_2_SO_4_. We attribute this to the presence of oxygen functionalities onto the graphene material obtained in LiClO_4_ which enable charge electrostatic interactions with the –NO_2_ groups of the analyte, in addition to π-π stacking interactions with the aromatic moiety. Research findings obtained from this study would assist in the development of portable devices for the on-site detection of nitroaromatic explosives.

Modern military explosives are generally nitrogen-containing aromatic compounds (NAC)^1^,^2^ that can undergo self-oxidation to release a sudden outburst of heat and potential energy^3^,^4^. With its widespread application in military explosives^2^,^5^ for the past 100 years^2^, 2,4,6-trinitrotoluene (TNT) is one of the most representative nitro-substituted aromatic explosive. In addition, the more volatile^6^,^7^ and soluble^8^,^9^ 2,4-dinitrotoluene (DNT) serves as an intermediate in the commercial manufacturing of TNT, thus it is often detected as an impurity^6^ indicating the presence of TNT in contamination sites. Unexploded ordnance and munitions may seep into groundwater^10^,^11^, which then enter and pollute the sea. These toxic contaminants eventually manifest as adverse health impacts in humans, such as discolouration of hair, skin and nails^12^,^13^, aplastic anaemia^14^,^15^ and liver function disturbances^15^, due to effects of bioamplification and bioaccumulation. With the prevalence of TNT in military munitions, the swift and accurate forensic detection of DNT and TNT NAC explosives are therefore imperative in ensuring national security and environmental protection.

Conventional analytical methods for the qualitative and quantitative analysis of DNT and TNT include gas chromatography^2^,^16^, high performance liquid chromatography^2^,^16^,^17^, Raman spectroscopy^2^,^7^,^18^, infrared absorption spectroscopy^2^,^19^, mass spectrometry^2^,^20^, immunoassay techniques^21^,^22^ and electrochemical techniques^17^,^23^,^24^. Coupled with advantages of high sensitivity^23^,^24^, large linear range^23^, low-cost instrumentation^10^,^23^, portability^11^ and short response time^10^,^11^, electrochemical methods offer the possibility of on-site, real-time analysis in comparison to other analytical techniques, which is crucial for the development of highly affordable and reliable devices for the *in situ* detection and measurement of NAC explosives. In a research paper by Dong *et al*. comparing the electrochemical and fluorescent detection methods for TNT analysis based on carbon quantum dots, a broader linear detection range was possible for the electrochemical sensor^25^. Electrochemical detection of DNT and TNT are made possible by the 4-electron stepwise reduction of each –NO_2_ group to an –NHOH group^6^ followed by the 2-electron reduction of –NHOH group to an –NH_2_ group^26^,^27^.

Since the successful isolation of graphene in 2004^28^, research interest in this one-atom-thick, two-dimensional carbon nanomaterial has risen exponentially owing to its extraordinary thermal^29^,^30^,^31^, mechanical^32^,^33^, optical^34^, electronic^35^,^36^ and electrochemical^37^,^38^ properties. Electron delocalisation within the large sp^2^ network of graphene promotes the adsorption of electron-deficient aromatic compounds onto the nanomaterial surface, via π-π electron donor-acceptor (EDA) stacking interactions^39^,^40^. As such, graphene is a suitable candidate for the trace electrochemical detection of NACs. Our research group has therefore investigated a wide range of graphene-based materials, such as graphene nanoribbons and nanosheets^5^,^41^, hydrogenated graphene^42^ as well as graphenes prepared from Hummers, Staudenmaier and Hofmann syntheses methods^11^,^41^,^42^ in order to isolate the most suitable probe material for the sensing of NAC explosives in seawater. Various works performed by other research groups have also supported the high selectivity and sensitivity of functionalised graphene, including graphene modified with Ag nanoparticles^43^, PtPd concave nanocubes^44^ and ionic liquid^45^, as well as other carbon-based electrochemical sensors such as carbon quantum dots for highly efficient NACs detection^25^.

Recently, a novel and highly efficient method has emerged as a possible avenue for the mass production of graphene: the electrochemical exfoliation of graphite under anodic or cathodic conditions, which enables the intercalation of electrolyte ions between the graphene layers, resulting in their exfoliation^46^. It has been previously established that the physicochemical properties of graphene is strongly affected by the exfoliation conditions such as the applied potential and electrolyte used for the procedure^47^. As such, we aim here to study the application of electrochemically exfoliated graphene prepared from different electrolytes for the detection of DNT and TNT in seawater. Graphene sheets are prepared from the electrochemical exfoliation of commercially available graphite foil using two different electrolytes under anodic conditions: LiClO_4_ and Na_2_SO_4_.

## Results

### Inherent Electrochemistry of Electrochemically Exfoliated Graphene Materials

**Figure 1:**
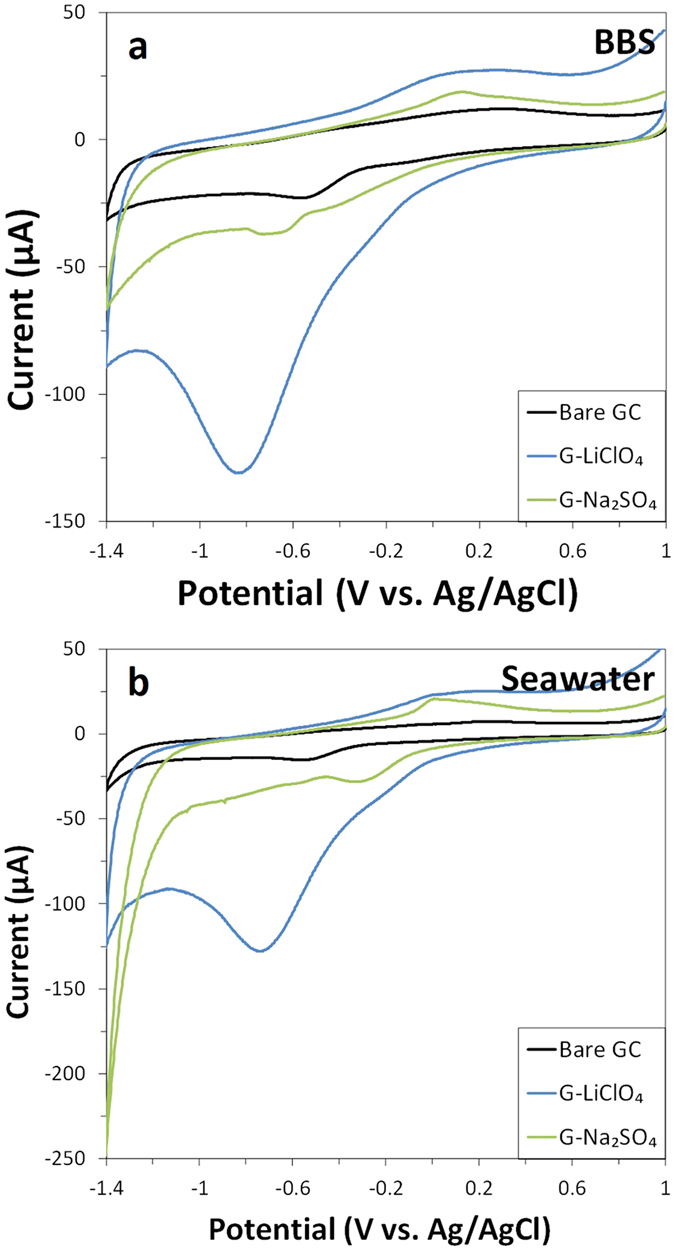
Cyclic voltammograms of (**a**) blank buffer and (**b**) seawater for GC electrodes modified with graphene obtained in LiClO_4_ (G-LiClO_4_) and Na_2_SO_4_ (G-Na_2_SO_4_). Cyclic voltammograms of bare GC electrode is also shown for comparison. Conditions: (**a**) BBS (20 mM, pH 9.2) and (**b**) mixture of 9:1 volume ratio of seawater (pH 8.0) to BBS (200 mM, pH 9.2). Scan rate 100 mV s^−1^.

Cyclic voltammograms were recorded in a blank buffer (borate buffer solution, BBS, pH 9.2) to ascertain the presence of inherent reduction peaks for the graphene materials that may interfere with the electrochemical reduction of DNT and TNT (Fig. 1a). Cyclic voltammetric measurements were also performed in seawater to determine the influence of species present in the matrix (Fig. 1b). The pH of all electrolytes were maintained within the pH range of 8 to 10, which had been previously established by our group to produce the largest and most stable current response for a variety of graphene-based materials^41^. Graphene prepared from LiClO_4_ (G-LiClO_4_) showed an intense, broad reduction peak at −819 mV and −724 mV in BBS and seawater respectively, whereas graphene synthesised in Na_2_SO_4_ (G-Na_2_SO_4_) showed a low-intensity peak at −655 mV in BBS which does not seem to appear when measuring in seawater. Overall, relatively similar voltammograms were obtained in both BBS and seawater within the 0 mV to −1000 mV potential range, which is indicative of the absence of electroactive compounds in seawater that may complicate the analysis of NAC explosives. The intense inherent reduction peak recorded for G-LiClO_4_ in both BBS and seawater can be attributed to the irreversible reduction of the oxygen functionalities present onto this material that can undergo electrochemical reduction. This phenomenon has been investigated more in detail and explained in previous reports^48^,^49^. Furthermore, the intensity of the reduction peak recorded for both graphene materials is in agreement with the corresponding C/O ratio which is of 4.0 for the G-LiClO_4_ and 8.8 for the G-Na_2_SO_4_ (see Supplementary Fig. S2).

### Electrochemical Detection of 2,4-Dinitrotoluene (DNT) and 2,4,6-Trinitrotoluene (TNT)

**Figure 2:**
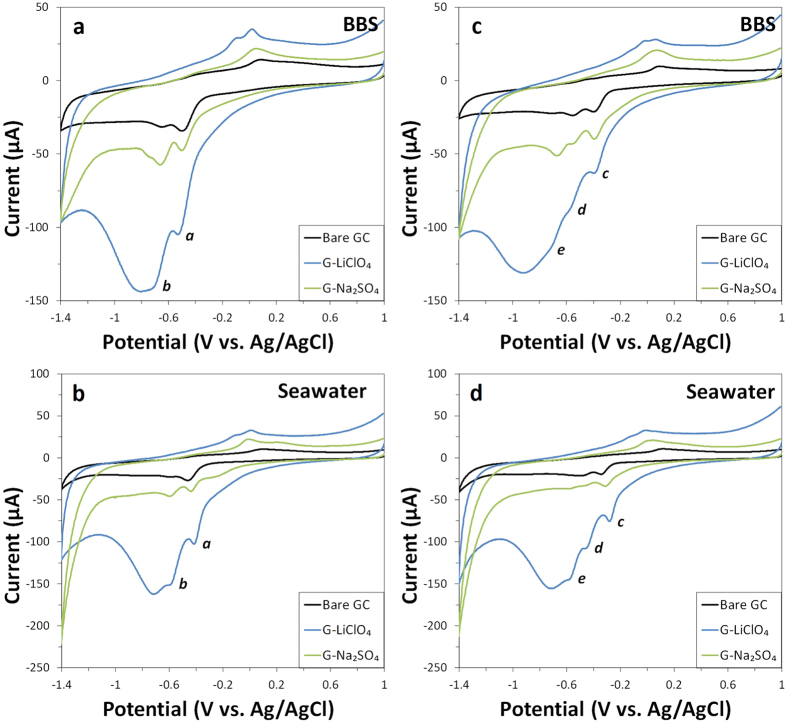
Cyclic voltammograms of DNT (20 ppm) (**a,b**) in (**a**) BBS and (**b**) seawater and TNT (20 ppm) in (**c**) BBS and (**d**) seawater at bare GC electrode and GC electrode modified with G-LiClO_4_ and G-Na_2_SO_4_. Conditions: (**a,c**) BBS (20 mM, pH 9.2) and (**b,d**) mixture of 9:1 volume ratio of seawater (pH 8.0) to BBS (200 mM, pH 9.2). Scan rate 100 mV s^−1^.

Cyclic voltammetry scans were first obtained in BBS to investigate the electrochemical behaviour of DNT and TNT on the electrochemically exfoliated graphenes and then repeated in seawater to determine the feasibility and reliability of real-time environmental analysis (Fig. 2).

The reduction of DNT revealed two major reduction peaks in both BBS and seawater systems, and they are labelled *a* and *b* by order of appearance in the CV cathodic wave (Fig. 2a,b). Notably, peak *b* coincides with the inherent reduction peak of oxygen functionalities on the graphene materials. As such, the first reduction peak – peak *a* – is conveniently chosen for the quantitative analysis of DNT. For all three electrode systems, the reduction potential of peak *a* was less negative in seawater than in BBS (Table 1). The peak height of the reduction wave *a* was generally comparable in both BBS and seawater systems.

Cyclic voltammograms of TNT revealed, as expected, three major reduction peaks in both BBS and seawater systems, labelled as *c, d* and *e* by order of appearance in the CV cathodic wave (Fig. 2c,d). Notably, peaks *d* and *e* are almost undetectable for bare GC and G-LiClO_4_ in BBS, and for bare GC and G-Na_2_SO_4_ in seawater. In addition, peak *e* coincides with the inherent reduction peak of oxygen functionalities for G-LiClO_4_. As such, the first reduction peak – peak *c* – is chosen for the quantitative analysis of TNT. Similar to the electrochemical sensing of DNT, peak *c* consistently appeared at lower reduction potentials for all three electrodes systems when switched from the BBS system to the seawater system (Table 1). The peak height of the reduction wave *c* was also generally comparable in both BBS and seawater systems, with the exception of G-LiClO_4_ displaying a marked increase in peak intensity in the seawater system.

**Figure 3:**
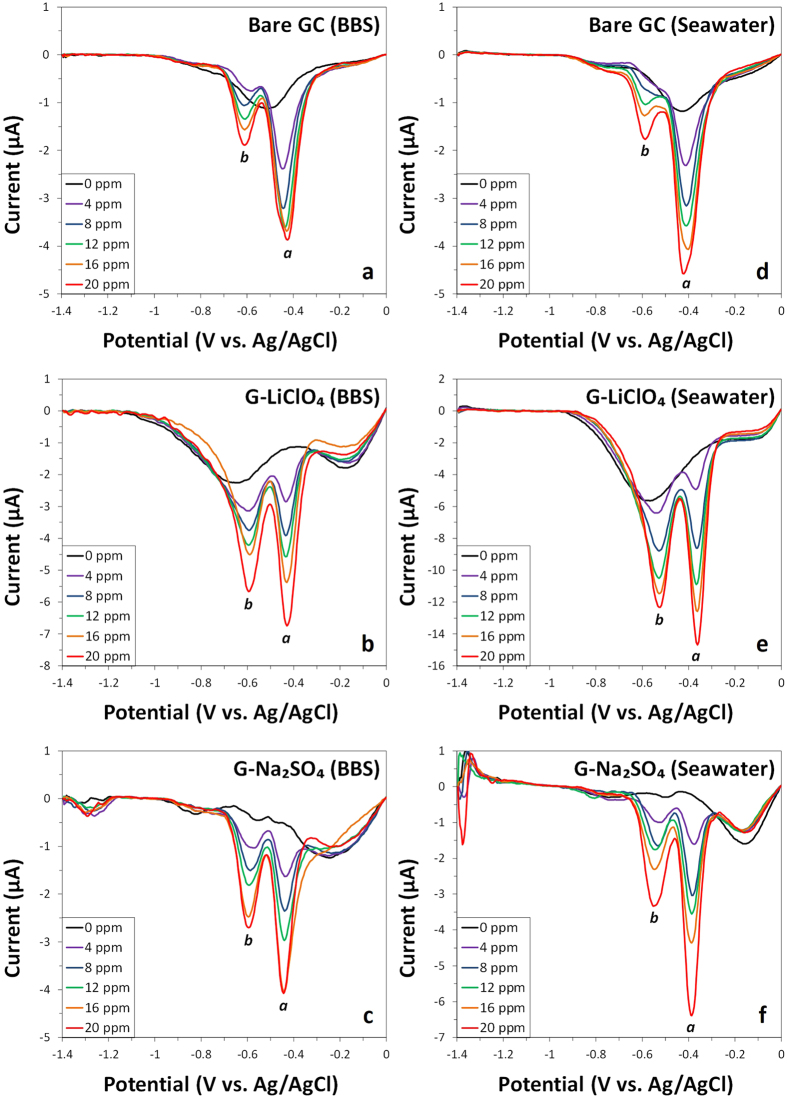
Differential pulse voltammograms (baseline corrected) of various concentrations of DNT (0, 4, 8, 12, 16 and 20 ppm) in BBS (left panel) and seawater (right panel) at bare GC electrode (**a,d**) and at electrodes modified with G-LiClO_4_ (**b,e**) and G-Na_2_SO_4_ (**c,f**). Conditions: (**a–c**) BBS (20 mM, pH 9.2) and (**d–f**) mixture of 9:1 volume ratio of seawater (pH 8.0) to BBS (200 mM, pH 9.2).

After the investigation carried out using cyclic voltammetry, a more sensitive electrochemical technique – differential pulse voltammetry – was then chosen for the trace analysis of DNT and TNT. As shown in Fig. 3, differential pulse voltammograms were obtained in BBS and seawater, for a range of DNT concentrations spanning across 0 ppm and 20 ppm, in increments of 4 ppm and using the optimized amount of electrocatalyst deposited on the GC electrode surface (see Supplementary Fig. S3). A larger signal response is apparent in the seawater system for both electrochemically exfoliated graphene materials, in particular with the G-LiClO_4_ demonstrating a doubling of current intensities at all concentrations, as compared to the BBS system (see Supplementary Fig. S4). In addition, G-LiClO_4_ exhibited the largest reduction peak intensities across all concentrations in both BBS and seawater systems, as compared to the other two electrode systems.

**Figure 4:**
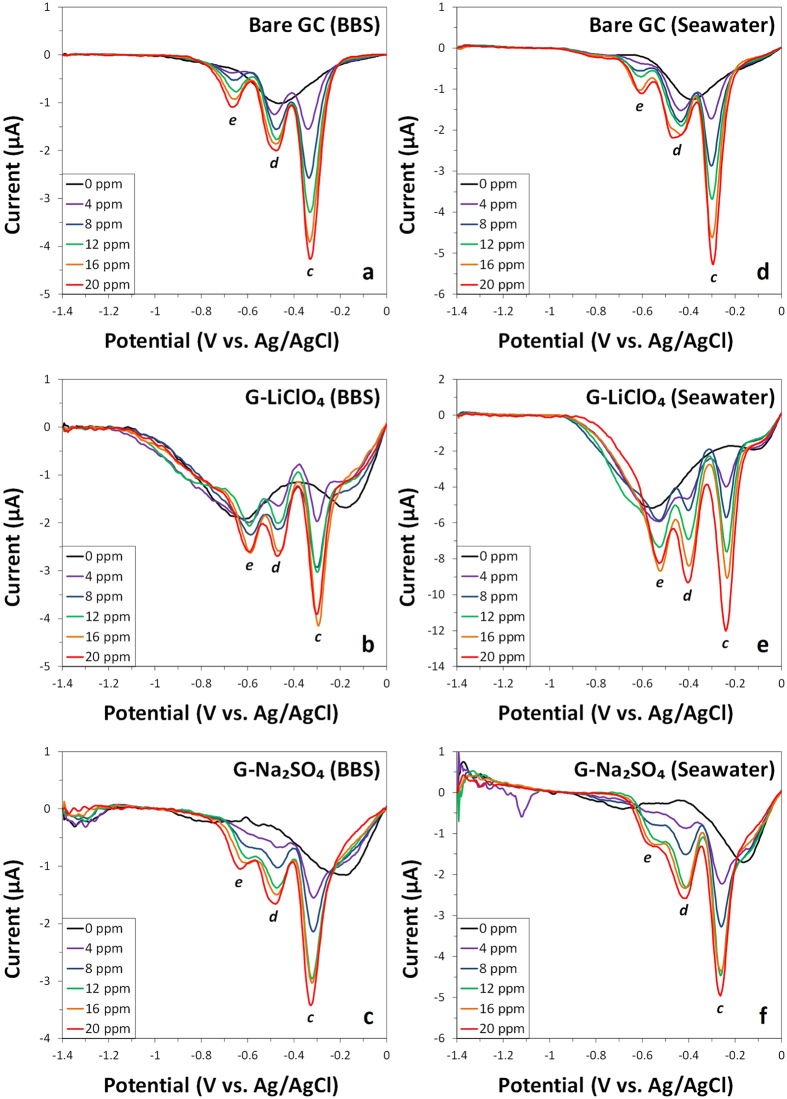
Differential pulse voltammograms (baseline corrected) of various concentrations of TNT (0, 4, 8, 12, 16 and 20 ppm) in BBS (left panel) and seawater (right panel) at bare GC electrode (**a,d**) and at electrodes modified with G-LiClO_4_ (**b,e**) and G-Na_2_SO_4_ (**c,f**). Conditions: (**a–c**) BBS (20 mM, pH 9.2) and (**d–f**) mixture of 9:1 volume ratio of seawater (pH 8.0) to BBS (200 mM, pH 9.2).

Differential pulse voltammetry study in BBS and seawater for TNT (Fig. 4) revealed a larger signal response in the seawater system for both electrochemically exfoliated graphene materials, with G-LiClO_4_ demonstrating an increase in current intensities by 2 folds at all concentrations, as compared to the BBS system (see Supplementary Fig. S5). In addition, G-LiClO_4_ exhibited the largest reduction peak intensities across all concentrations in both BBS and seawater systems.

**Figure 5:**
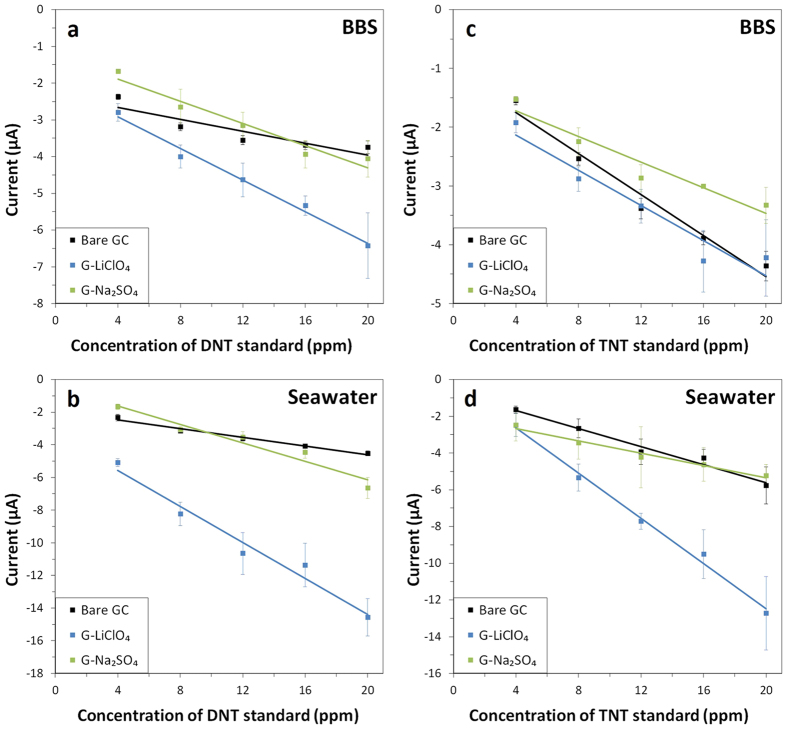
Concentration dependence of DNT (**a,b**) in (**a**) BBS and (**b**) seawater and TNT (**c,d**) in (**c**) BBS and (**d**) seawater at bare GC electrode and GC electrode modified with G-LiClO_4_ and G-Na_2_SO_4_. Conditions: (**a,c**) BBS (20 mM, pH 9.2) and (**b,d**) mixture of 9:1 volume ratio of seawater (pH 8.0) to BBS (200 mM, pH 9.2). Data is based on the first reduction peak of DNT and TNT.

Calibration graphs based on the current intensities of reduction peak *a* obtained from DPV measurements were then plotted as a linear function of spiked DNT concentrations (Fig. 5a,b).

The overall sensitivity of the electrode system for DNT detection, represented by the slopes of the linear calibration plots, was highest for the G-LiClO_4_, followed by G-Na_2_SO_4_, then the bare GC electrode, in both BBS and seawater systems. G-LiClO_4_ consistently demonstrated higher current intensities than the other two electrode systems at all DNT concentrations. The LOD value was lowest for G-LiClO_4_, followed by G-Na_2_SO_4_, then bare GC in BBS buffer. In the seawater system, the LOD value was lowest for bare GC, followed by G-LiClO_4_, then G-Na_2_SO_4_ (Table 2).

It is noteworthy that both graphene materials exhibited enhancements in sensitivities when switched from the BBS system to the seawater system. In particular, doubling of system sensitivity was achieved for G-LiClO_4_.

The calibration graphs based on reduction peak *c* plotted as a linear function of spiked TNT concentration (Fig. 5c,d) showed that the sensitivity of the electrode system was highest for bare GC, followed by G-LiClO_4_, then G-Na_2_SO_4_ in BBS buffer. When measuring in seawater, a different result was obtained. Electrode modified with G-LiClO_4_ consistently demonstrated higher current intensities than the other two electrode systems at all TNT concentrations. The LOD value was lowest for bare GC, followed by G-Na_2_SO_4_, then G-LiClO_4_ in BBS buffer. In the seawater system, the LOD value was lowest for G-LiClO_4_, followed by bare GC, then G-Na_2_SO_4_.

It is noteworthy that all three electrode systems exhibited enhancements in sensitivities in the seawater system as compared to the BBS system. In particular, tripling of system sensitivity was achieved for G-LiClO_4_. Improvements in electrochemical detection of TNT by G-LiClO_4_ in comparison to G-Na_2_SO_4_ were also observed similar to DNT sensing.

The enhanced electrochemical performance of G-LiClO_4_ as compared to G-Na_2_SO_4_ demonstrates that π-π stacking interactions cannot be considered as the sole factor influencing the electron transfer behaviour of the graphene materials towards the reduction of NACs. Evidently here the presence of oxygen functional groups are beneficial to the adsorption and electrochemical reduction of NACs despite a less efficient π-π stacking interaction due to the competition with water molecules^50^ and also a reduced conductivity of graphene materials carrying oxygen moieties^51^. As demonstrated in other works^52^,^53^, concomitant factors influence the electroanalytical detection of redox active molecules with the dominant one differing in relation to each particular analyte. A careful investigation and optimisation is thus highly recommended prior the application of newly prepared carbon materials for electrochemical sensing purposes.

The improved electrochemical signal observed for G-LiClO_4_ may be the result of additional electrostatic interactions between the electron-withdrawing –NO_2_ groups of NACs and the oxygen functionalities on the graphene material^54^, dominating over the EDA stacking interactions. This study highlights the possibility of using graphene-based nanomaterials with high oxygen content as potential electrode material for the on-site electrochemical detection of nitro-substituted aromatic explosives.

**Figure 6:**
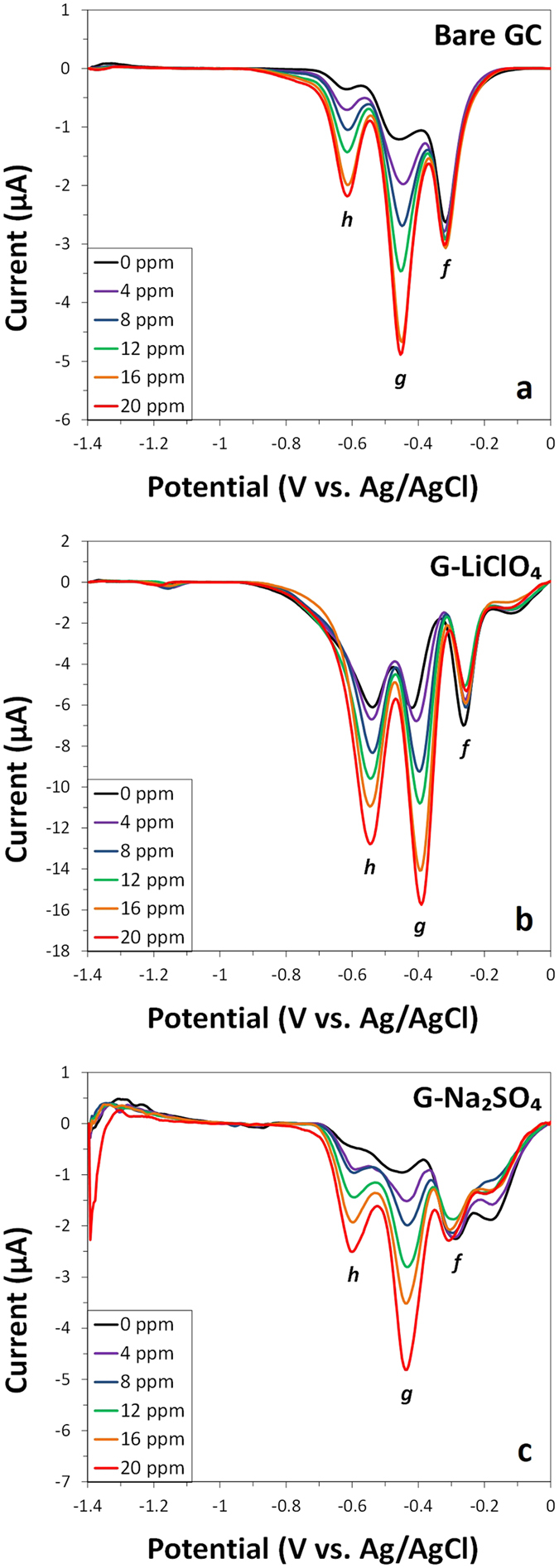
Differential pulse voltammograms (baseline corrected) of various concentrations of DNT (0, 4, 8, 12, 16 and 20 ppm) in seawater containing TNT (8 ppm) at (**a**) bare GC electrode and at electrodes modified with (**b**) G-LiClO_4_ and (**c**) G-Na_2_SO_4_. Conditions: mixture of 9:1 volume ratio of seawater (pH 8.0) to BBS (200 mM, pH 9.2).

The selectivity of this sensing platform was evaluated in the seawater system, by observing changes in electrochemical response upon spiking increasing amounts of DNT into a solution containing 8 ppm of TNT. The simultaneous reduction of DNT and TNT revealed three major reduction peaks, which are denoted as *f, g* and *h* by order of appearance in the cathodic wave (Fig. 6). As it can be seen in Fig. 6, the reduction peak *f* can be solely assigned to TNT since it remains fairly consistent with the increase of DNT concentration and it is clearly independent. This allows a selective detection of TNT in the presence of DNT. In addition, peak g and h follow closely to the reduction signals recorded for DNT with negligible influence from the second and third reduction waves produced by TNT. This phenomenon is even more pronounced for G-LiClO_4_ due to the enhanced electrochemical performance of the material. The sensing platform proposed in this work can thus selectively detect DNT and TNT in a NAC mixture sample.

## Conclusions

This study has investigated and compared the sensing abilities of electrochemically exfoliated graphene obtained from the anodic exfoliation of graphite foil in different electrolytes (LiClO_4_ and Na_2_SO_4_), for the detection of nitro-substituted aromatic explosives (DNT and TNT) in both borate buffer and seawater. Voltammetry studies reveal that G-LiClO_4_ which carries the highest amount of oxygen functional groups, is the most suitable electrode system for the detection of NAC explosives, due to a favourable electrostatic interaction with the analytes which resulted in a higher sensitivity and linearity. On the other hand, G-Na_2_SO_4_ consistently demonstrates similar electrochemical performance with the unmodified GC electrode for NAC detection. Significant improvements in electrode system sensitivity and linearity for DNT and TNT detection in seawater as compared to BBS were also observed for G-LiClO_4_, accompanied by the ability of our sensing platform to distinguish between DNT and TNT, which supports the applicability and effectiveness of using G-LiClO_4_ in real-time environmental analysis of seawater.

## Methods

### Materials

Sodium tetraborate decahydrate and 2,4-dinitrotoluene (DNT) were purchased from Sigma-Aldrich. *N*,*N*-dimethyl formamide (DMF) and acetonitrile were purchased from Merck (Singapore). 2,4,6-trinitrotoulene (TNT) was purchased from AccuStandard (New Haven, CT) as an analytical standard in the diluted form (1000 ppm in acetonitrile). Graphene electrochemically exfoliated in LiClO_4_ (G-LiClO_4_) and Na_2_SO_4_ (G-Na_2_SO_4_) were prepared and characterised in detail as previously reported^47^. The graphene flakes had an average size of 3–10 μm and an average thickness of 2.8 nm. G-LiClO_4_ had a D/G ratio of 1.00 and a C/O ratio of 4.0, whereas G-Na_2_SO_4_ had a D/G ratio of 0.95 and a C/O ratio of 8.8. Scanning transmission electron micrographs, XPS and Raman spectra are presented in the Supporting information file as Figs S1 and S2. Sand-filtered seawater (pH 8.0) was purchased from a local aquarium store in Singapore. Milli-Q water of resistivity 18.2 MΩ cm was used throughout the experiments.

### Apparatus

Voltammetry analyses were conducted at room temperature using a μAutolab Type III electrochemical analyser instrument (Electrochemie, Utrecht, The Netherlands) and controlled by General Purpose Electrochemical Systems Version 4.9 software. The experiments were performed in an electrochemical cell using a three-electrode system. Glassy carbon (GC, 3 mm in diameter) working electrode, platinum auxiliary electrode and Ag/AgCl (saturated) reference electrode were obtained from CH Instruments (Austin, TX).

### Electrochemical measurements

Suspensions (1 mg mL^−1^) of the electrochemically exfoliated graphene materials were prepared from the dispersion of graphene flakes in DMF and sonication for 2.5 h. An aliquot (1 μL) of the suspension was then drop-casted onto a GC electrode surface that had been polished with alumina particles (0.05 μm) on a polishing pad, and allowed to evaporate under a heat lamp. The deposition step was performed to obtain one to three layers of graphene material on the GC electrode for the optimisation experiments. DNT was prepared as a stock solution (1000 ppm in acetonitrile). TNT (1000 ppm in acetonitrile) was used as received. Borate buffer (BBS, pH 9.2) was prepared from dissolving Na_2_B_4_O_7_.10H_2_O in milli-Q water. Seawater (pH 8.0) was mixed with BBS (200 mM, pH 9.2) in a ratio of 9:1 prior to use. All cyclic voltammetry (CV) measurements were performed with a scan rate of 100 mV s^−1^. All differential pulse voltammetry (DPV) measurements were performed with a modulation amplitude of 25 mV and a step potential of 5 mV. All voltammetry measurements were repeated three times to ensure measurement reproducibility, with the most representative voltammogram being selected for data presentation. Voltammetry measurements were also conducted on the unmodified GC electrode for reference purposes. Degassing was not carried out prior to electrochemical measurements to simulate real-time environmental analysis.

## Additional Information

**How to cite this article**: Yew, Y. T. *et al*. Nitroaromatic explosives detection using electrochemically exfoliated graphene. *Sci. Rep.* **6**, 33276; doi: 10.1038/srep33276 (2016).

## Supplementary Material

Supplementary Information

## Figures and Tables

**Table 1:** Reduction peak potentials (mV) of DNT (20 ppm) and TNT (20 ppm) in BBS and seawater at bare GC electrode and electrode modified with G-LiClO_4_ and G-Na_2_SO_4_.

**Material**	**DNT**		**TNT**		
	**Peak** ***a***	**Peak** ***b***	**Peak** ***c***	**Peak** ***d***	**Peak** ***e***
**BBS**					
Bare GC	−492	−645	−389	−555	−702
G-LiClO_4_	−528	−694	−394	−567	−714
G-Na_2_SO_4_	−501	−660	−392	−543	−670
**Seawater**					
Bare GC	−462	−606	−343	−499	—
G-LiClO_4_	−416	−580	−279	−440	−565
G-Na_2_SO_4_	−435	−594	−311	−465	−587

**Table 2:** Sensitivities (μA ppm^−1^), LOD (ppm) and *R* ^2^ of various electrode systems towards DNT and TNT detection in (A) BBS and (B) seawater.

**Material**	**DNT**			**TNT**		
	**Sensitivity (μA ppm**^**−1**^)	**LOD (ppm)**	***R***^**2**^	**Sensitivity (μA ppm**^**−1**^)	**LOD (ppm)**	***R***^**2**^
**BBS**						
Bare GC	−0.081	11.26	0.820	−0.174	4.08	0.972
G-LiClO_4_	−0.215	2.73	0.987	−0.150	6.74	0.927
G-Na_2_SO_4_	−0.151	5.43	0.951	−0.109	6.54	0.931
**Seawater**						
Bare GC	−0.133	3.17	0.983	−0.246	3.76	0.976
G-LiClO_4_	−0.552	4.35	0.968	−0.617	2.03	0.993
G-Na_2_SO_4_	−0.284	5.97	0.942	−0.167	3.85	0.975

Data is based on the first reduction peak of DNT and TNT.
