# Supplementary material for: Nitroaromatic explosives detection using electrochemically exfoliated graphene
Source: Sci Rep. 2016 Sep 16;6:33276. doi: 10.1038/srep33276 (PMC5025880; doi:10.1038/srep33276)
Supplement: Supplementary Information [file srep33276-s1.doc]

**Supporting information**

**Nitroaromatic explosives detection using electrochemically exfoliated graphene**

# Ying Teng Yew, Adriano Ambrosi, and Martin Pumera^*^

Division of Chemistry and Biological Chemistry, School of Physical and Mathematical Sciences, Nanyang Technological University, 21 Nanyang Link, Singapore 637371 (Singapore)

*E-mail: pumera@ntu.edu.sg

**Characterisation of graphene materials**

**Figure S1**. Scanning transmission electron micrograph of graphene materials exfoliated in A) Na_2_SO_4_ and B) LiClO_4_. Scale bars correspond to 1 μm.

High resolution XPS (C1s) scans uncovered the presence of C−O, C=O, and O−C=O peaks, reflecting the presence of hydroxyl, epoxy, carbonyl and carboxylic functionalities in both G-Na_2_SO_4_ and G-LiClO_4_. According to XPS, graphene exfoliated in LiClO4 possesses a higher content of hydroxyl and carbonyl groups.

A

B

**Figure S2**. A) XPS and B) Raman spectra recorded for graphene exfoliated in Na_2_SO_4_ (top) and in LiClO_4_ (bottom). Raman spectra are recorded in correspondence to the marks in the optical images (right panel). (adapted from ref [36]).

**Optimisation of Electrocatalyst Mass**

The sensitivities of the electrode systems were highest for 1 µg of electrocatalyst deposited on the GC surface for both graphene materials. A coating mass of 1 µg was therefore selected as the optimal mass for voltammetry experiments in this paper.

A

B

**Figure S3.** Reduction peak intensities (µA) based on triplicate differential pulse voltammetry measurements for DNT (4, 8 and 12 ppm) at GC electrode modified with different masses of (A) G-LiClO_4_ and (B) G-Na_2_SO_4_. Conditions: BBS (20 mM, pH 9.2). Data is based on the first reduction peak of DNT.

**Current intensities of reduction peak *a* for DNT (20 ppm)**

A larger signal response is observed in the seawater system for both electrochemically exfoliated graphene materials. In particular, the graphene prepared in LiClO_4_ exhibits an increase in current intensity by 127% in the seawater system, as compared to the BBS system. On the other hand, bare GC and graphene prepared in Na_2_SO_4_ exhibit much smaller increments in current intensities by 21% and 64% respectively. In addition, graphene prepared using LiClO_4_ demonstrated the highest current intensity among all three electrode systems, giving an enhancement in peak intensity by 72% in BBS and 222% in seawater when compared to bare GC. Graphene prepared using Na_2_SO_4_ resulted in much smaller enhancement in electrochemical performance to that of bare GC, giving an enhancement in peak intensity by only 8% in BBS and 47% in seawater when compared to bare GC.

**Figure S4.** Reduction peak intensities (µA) based on triplicate differential pulse voltammetry measurements for DNT (20 ppm) at bare GC electrode and at electrodes modified with G-LiClO_4_ and G-Na_2_SO_4_. Conditions: BBS (20 mM, pH 9.2), or mixture of 9 : 1 volume ratio of seawater (pH 8.0) to BBS (200 mM, pH 9.2). Data is based on the first reduction peak of DNT.

**Current intensities of reduction peak *c* for TNT (20 ppm)**

A larger signal response is observed in the seawater system for both electrochemically exfoliated graphene materials. In particular, the graphene prepared in LiClO_4_ exhibits an increase in current intensity by 201% in the seawater system, as compared to the BBS system. On the other hand, bare GC and graphene prepared in Na_2_SO_4_ exhibit much smaller increments in current intensities by 32% and 57% respectively. In the BBS system, graphene prepared using LiClO_4_ and bare GC demonstrated comparable electrochemical performances, with a difference in peak intensities of only 3%. However, in the seawater system, graphene prepared using LiClO_4_ demonstrated the highest current intensity among all three electrode systems, giving an enhancement in peak intensity by 121% when compared to bare GC. Graphene prepared using Na_2_SO_4_ resulted in poorer electrochemical performance than bare GC, giving a lowering in peak intensity by 24% in BBS and 9% in seawater when compared to bare GC.

**Figure S5.** Reduction peak intensities (µA) based on triplicate differential pulse voltammetry measurements for TNT (20 ppm) at bare GC electrode and at electrodes modified with G-LiClO_4_ and G-Na_2_SO_4_. Conditions: BBS (20 mM, pH 9.2), or mixture of 9 : 1 volume ratio of seawater (pH 8.0) to BBS (200 mM, pH 9.2). Data is based on the first reduction peak of TNT.
